# The application of central tension plate with sharp hook in the treatment of intra-articular olecranon fracture

**DOI:** 10.1186/1471-2474-14-308

**Published:** 2013-10-28

**Authors:** Wei Chen, Qi Zhang, Zhiyong Hou, Yingze Zhang

**Affiliations:** 1 Department of Orthopaedic Surgery, The Third Hospital of Hebei Medical University, 050051 Shijiazhuang, Hebei, People’s Republic of China

**Keywords:** Olecranon, Fracture, Plate fixation, Central tension plate

## Abstract

**Background:**

Standard plate fixation can be used to treat intraarticular olecranon fractures with satisfactory functional recovery, but its use is accompanied by implant related complications. This retrospective study reports on the functional outcome of intraarticular olecranon fractures treated with a central tension plate with sharp hook.

**Methods:**

A retrospective review of any patient with an olecranon fracture from August 2007 to December 2008 was conducted. Patients were considered for inclusion in the study if they were treated surgically with a central tension plate with sharp hook. Patients with pathological fractures or previous fractures of the proximal ulna were excluded. The quality of reduction was evaluated using postoperative imaging. The functional recoveries of the affected upper limbs were evaluated postoperatively at regular intervals using the Mayo Elbow Performance (MEP) score and Disability of the Arm, Shoulder and Hand questionnaire (DASH).

**Results:**

Twenty six patients met the study criteria and were included in analysis. There were ten Type IIA, nine Type IIB, four Type IIIA and three Type IIIB fractures according to the Mayo classification system. Thirteen patients exhibited other concomitant fractures at the time of surgery: one patient with a coronoid fracture, two with a fracture of the radial head, and ten with fractures in other bones. Postoperative radiographic assessment revealed an anatomical or nearly anatomical reduction of all olecranon fractures treated. All olecranon fractures healed at an average of 14 weeks (range, 9 to 32 weeks). The patients were followed up for 42 months (range, 32 to 54 months). The mean DASH score was 8.5 (range, 0 to 31.7). The mean MEP score was 93.6 (range, 75 to 100). Based on the MEP score, all patients achieved good or excellent outcomes. No symptomatic plate removal was performed at the time of last follow-up.

**Conclusion:**

The central tension plate with sharp hook closely contours to the osteology of the proximal ulna. Treating intra-articular olecranon fracture with this new plate can achieve good to excellent functional outcome with a high union rate and a low incidence of hardware related complications.

## Background

Olecranon fractures are among the most common injuries of the upper extremity [1]. They make up approximately 10% of all fractures of the adult elbow and they range from simple nondisplaced fractures to complex fracture-dislocations of the elbow [2]. These fractures are commonly intraarticular, except for avulsion fractures of brachial triceps. Therefore, in order to avoid arthritis of the elbow joint, careful anatomical reduction by internal fixation is typically required for any intraarticular olecranon fracture. Tension band wiring (TBW) has been considered as the gold standard fixation to treat displaced transverse intraarticular olecranon fractures [3]. However, TBW fixation has demonstrated a high incidence of reoperation for the removal of symptomatic hardware [4-8], and thus internal fixation of comminuted olecranon fractures has evolved toward the use of more stable constructs [9-11]. As such, plate fixation has gradually gained popularity. Plate fixation is reported to give adequate stability and achieve fracture union in both simple and comminuted olecranon fractures [12]. The olecranon plate can be placed either laterally or posteriorly [12]. However, some plates don’t contour well to the osteology of the proximal ulna, which may necessitate hardware removal because of their prominence [13]. With this problem in mind, the central tension plate with sharp hook was engineered to reduce the risk of complications secondary to poor anatomic congruency, and thus improve the clinical outcome. We conducted this retrospective study to introduce the surgical technique for olecranon fractures treated with the central tension plate with sharp hook and we present the preliminary results with a minimum follow-up of 32 months.

## Methods

### Patients

A retrospective analysis of the patient database was conducted to identify the olecranon fractures that were treated with central tension plates with sharp hook at a single surgical center from August 2007 to December 2008. Patients were considered for inclusion in the study if they met the following criteria: age at least 18 years or older, underwent surgery for an olecranon fracture and a central tension plate with sharp hook was used, and if they were followed up for more than 12 months. Patients were excluded from this study if they sustained pathological fractures or previous fractures of the proximal ulna.

Prior to surgery, all patients were educated regarding the central tension plate, and informed consent was obtained from each patient. The Institutional Review Board of the Third Hospital of Hebei Medical University approved the study after thorough examination and verification.

### The structure of central tension plate with sharp hook

**Figure 1:**
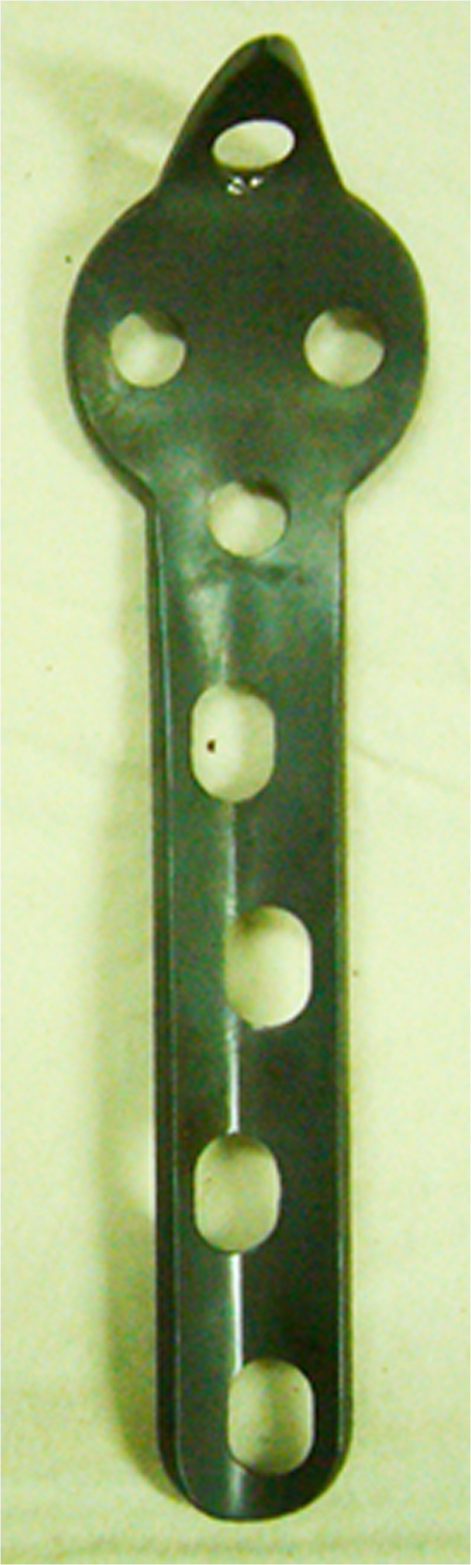
The anterior view of the central tension plate with sharp hook.

The central tension plate with sharp hook has obtained the Certificate of Invention Patent (Certificate No. 649355, Patent No. ZL 2008 1 0079748.X). Distally to proximally, the plate consists of a low profile angle-plate shaped body, then a gourd-shaped component, and finally a sharp hook (Figure 1). The plate is placed on the dorsal surface of the proximal ulna rather than the lateral surface. The angle of the plate body changes gradually from 110 degrees proximally to 80 degrees distally, which corresponds with the anatomical morphology of the ulna crest [14]. The gourd-shaped proximal component of the plate is designed specially to contour to the olecranon. There are three holes in the proximal component of the plate, which are used to permit multiple-angle insertion of screws to repair comminuted fragments. The central tension plates used in the current study are not locking ones.

### Operative technique

A sterile tourniquet is placed on the upper arm after skin preparation and draping. A longitudinal posterior skin incision is made to expose the olecranon. The dorsal surface of the proximal ulna is exposed far enough to accommodate the plate. If present, any impacted articular fragment is elevated and any coronoid fracture is reduced and provisionally fixed to the ulna with one or two Kirschner wires. After primary reduction and provisional fixation of the olecranon fracture, the plate is placed on the dorsal tension surface of the proximal ulna. The proximal component of the plate matches the contour of the olecranon. The sharp hook is inserted into the triceps tendon just over the tip of the olecranon. The plate is held in position and screw holes distal to the fracture line are drilled, measured, and tapped. Cortical screws are inserted into the oval plate holes but not fully tightened to permit sliding of the plate to compress the fracture fragments. The trajectories for the cortical screws are slightly medial or lateral to the central line of the plate to avoid entering the proximal radioulnar joint and to leave room for an axial cancellous screw, which is later inserted through the most proximal hole along the shaft of the ulna. The fracture is then compressed with the insertion of long intramedullary cancellous screws and the cortical screws distal to the fracture line are then tightened to secure the plate to the ulna. The subcutaneous tissues and skin are closed in the usual manner. Finally, a removable splint is applied with the elbow flexed to 90 degrees.

### Rehabilitation and postoperative evaluation

Active motion of the fingers and isometric contraction of the upper arm muscles is recommended as soon as pain can be tolerated. Gentle passive and active-assisted motion is initiated at 2 to 3 days postoperatively. It is recommended that patients take the arm out of the splint several times daily in order to exercise. Patients are instructed to gently flex and extend the affected elbow using the opposite hand, gradually increasing the range of motion as tolerated. Passive stretching and strengthening under occupational therapist supervision can be started at 6 weeks.

Follow ups were done and radiographic assessments were routinely performed at 4 weeks, 8 weeks, 12 weeks, 6 months, 12 months, and thereafter at a half-year or a 1-year interval. At each follow up appointment, the Mayo Elbow Performance (MEP) score and Disability of the Arm, Shoulder and Hand questionnaire (DASH) were completed. Measurements of elbow flexion, extension, and forearm rotation were done using a 1404. Hammer angle gage goniometer (Sanfeng Co. Weihai, China).

### Statistic analysis

All data were analyzed using SPSS 11.0 for Windows (SPSS Inc., Chicago, IL, USA), and descriptive summaries of the data were performed. Student’s *t* tests were used when comparing the scores between unaffected and affected limbs. Any difference with a P value of less than 0.05 was regarded as statistically significant.

## Results

**Figure 2:**
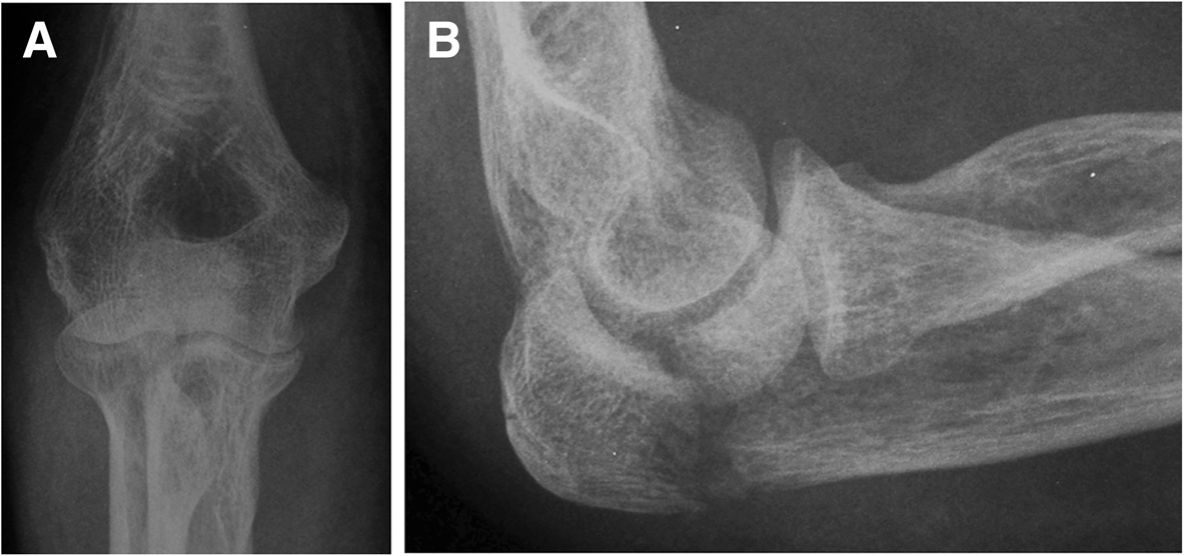
**A female patient sustained right displaced olecranon fracture which is a type IIA fracture according to the Mayo classification.** (**A**, the anteroposterior view. **B**, the lateral view).

**Figure 3:**
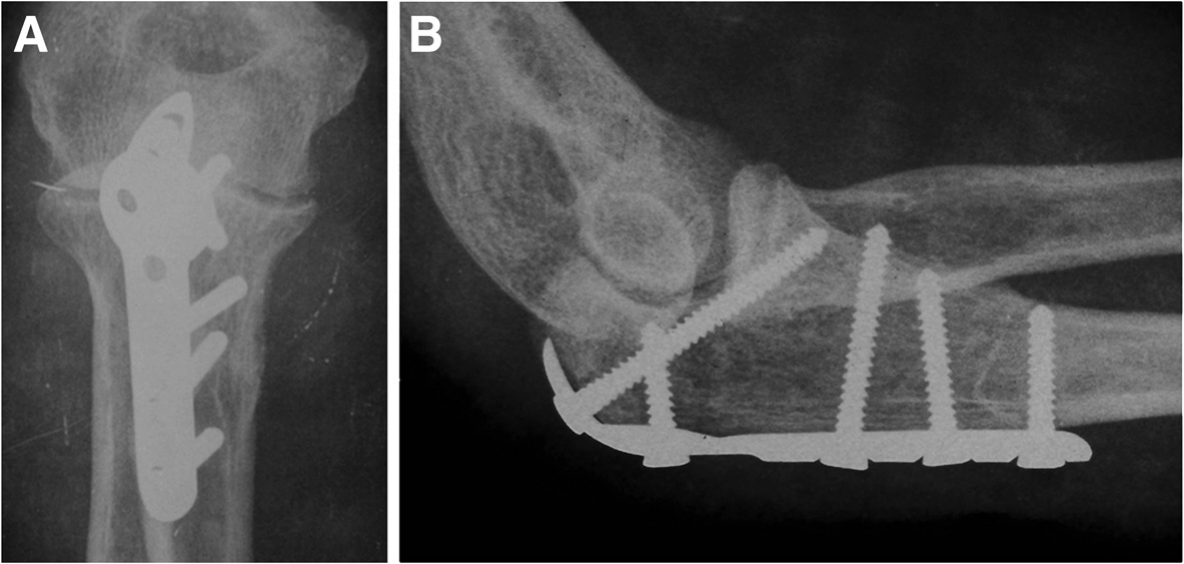
**Postoperative radiographic assessment demonstrated nearly anatomical reduction of the olecranon fractures.** (**A**, the anteroposterior view. **B**, the lateral view).

Twenty six patients were identified from the patient database and included into this study. The study group consisted of 16 men and 10 women with a mean age of 39.8 years (range, 19.2 to 74.5 years). There were 11 left and 15 right olecranon fractures. Using the Mayo classification [15], there were 10 Type IIA, 9 Type IIB, 4 Type IIIA and 3 Type IIIB fractures (Figure 2). Mechanisms of injury included 11 slips, 7 falls from bicycles or height of more than 3 meters, and 8 traffic accidents. None of the fractures were open injuries. Ten patients sustained other fractures at the time of olecranon injury, including two radial head fractures, one coronoid fracture, one lumbar fracture, one pelvic fracture, three femoral fractures and five tibial and fibular fractures. Patients were operated on an average of 2.3 days (range, 1 to 5 days) from the time of initial injury. The mean duration of operative time was 78 minutes (range, 55 to 135 minutes). The average blood loss was 74 mL (range, 40 to 200 mL). Postoperative radiographic assessment demonstrated anatomical or nearly anatomical reduction of olecranon fractures in all patients (Figure 3). No patients showed an articular gap or step of more than 2 mm postoperatively. All olecranon fractures in this series went on to heal at an average of 14 weeks (range, 9 to 32 weeks), without malunion, nonunion or soft tissue related complications.

The patients were followed up for an average of 42 months (range, 32 to 54 months). At the most recent follow up, no loss of reduction was noted in any patient. Measurements of elbow flexion, extension, and forearm rotation were recorded for both the affected and unaffected upper limbs. Only the elbow extension showed a small but statistically significant decrease (Table 1, P < 0.05). The evaluation of functional recovery of the affected elbow was performed. The mean DASH score was 8.5 (range, 0 to 31.7). The mean MEP score was 93.6 (range, 75 to 100). Mild pain due to prominent hardware was noted in one patient, but it was well tolerated and no patients requested plate removal during the follow-up period. Radiographic evidence of degenerative changes of the elbow joints had not been observed in any patient at the most recent follow-up appointment.

**Table 1:** The range of motion of the affected and unaffected elbows in 26 patients

	**Affected**	**Unaffected**	***P***
flexion (Mean ± SD, degrees)	138 ± 10	141 ± 6	0.105
extension (Mean ± SD, degrees)	7 ± 4	9 ± 2	0.014
pronation (Mean ± SD, degrees)	78 ± 8	80 ± 5	0.137
supination (Mean ± SD, degrees)	75 ± 7	78 ± 6	0.078

## Discussion

The current study demonstrates that anatomical or nearly anatomical reduction and satisfactory fixation of an olecranon fracture was obtained in all 26 patients treated with a central tension plate with a sharp hook. No fixation failures were reported. At the latest follow-up, no patient required plate removal secondary to symptomatic hardware complications. The range of motion of the injured elbow was greatly improved from the earlier postoperative time, and according to MEP and DASH scores, satisfactory functional recovery was achieved in all patients. The indications for central tension plate fixation include displaced unstable oblique and comminuted olecranon fractures. This technique is also suitable for transverse olecranon fractures in high-demand patients.

Early range of motion has been considered a critical aspect in postoperative care of olecranon fractures [16]. Restoration of articular congruity and rigid internal fixation are therefore essential in the treatment of intra-articular fractures, as they permit early postoperative range of motion. The challenge for these fractures, however, is that because of the subcutaneous nature of the proximal ulna, hardware prominence is common. Hardware prominence often causes discomfort to the patient, and is a reason to necessitate its removal. Indeed, prominent hardware requiring removal remains one of the most common complications following internal fixation of olecranon fractures [2], and up to 20% of plates have required removal to manage patient reported symptoms of discomfort [12,13]. Similarly, 80% of TBW fixations reportedly are removed because of migration and painful irritation [4-8,17]. Knowing that hardware prominence is such a common surgical complication, the central tension plate was designed to have a low profile, and the proximal component is in the shape of a gourd in order to better match the olecranon osteology. The sharp hook is inserted into the triceps tendon and positioned closely to the dorsal surface of proximal ulna. In the present case series, no symptomatic hardware removal was required. Mild pain over the elbow was noted in one patient, however, it was felt to be a result of the prominent end of a single screw.

To position a standard plate properly on the posterior surface of the ulna, it has been recommended that the triceps fascia and tendon be partially split, allowing the implant to rest directly on the bone [2]. There is the risk, however, that by splitting the tendon and fascia the triceps muscle strength of the operative extremity will be impaired, even if the tendon is sutured and reattached to the ulna once the plate is in place [18]. Using the central tension plate, the sharp hook can be directly inserted into the olecranon through the tendon of triceps muscle without making an incision. This may result in less injury to the triceps muscle than as seen secondary to routine posterior plating. It has been found that patients with isolated olecranon fractures typically lose 10° to 15° of extension, and this deficit is even greater when there is an associated fracture of the radial head or coronoid [2]. The data from the present study demonstrated that at follow up the range of motion of the affected elbow could return to near preoperative values, as the flexion and rotation of the affected elbows were similar to the unaffected ones, and the extension of the affected elbow was on average only 2 degrees less than the contralateral uninjured elbow.

Posterior plating is commonly used to manage olecranon fractures, as it facilitates fracture reduction [19] and is stronger than medial or lateral plating [20]. Gordon et al. reported that a posterior plate on the dorsal surface of proximal ulna with an intramedullary screw was significantly stronger than even dual medial and lateral plating [19]. In our study, all plates were placed on the dorsal surface of the ulna, which can improve the rigidity of fixation. The shape of plate also influences the rigidity of fixation. Reconstruction and one-third tubular plates may not resist saggital plane bending forces in those fractures with intercalary comminution, bone loss, concomitant radial oblique fractures or radial head subluxation [11]. In these situations, a stiffer implant should be considered. The body of the newly designed plate is v-shaped. It is known that a v-shaped construct is stronger than tubular constructs, and can better resist the saggital plane bending forces. At follow up, no loss of reduction was observed. Rigid internal fixation permits early exercises, and good or excellent functional recovery of the elbow was achieved in all patients in this case series.

There are limitations to this study, in particular its retrospective nature and the small number of patients treated with the central tension plate with sharp hook. As this study only reports the results of those patients treated with the central tension plate, a randomized controlled study with a control group of those patients treated with other commonly used plates or TBW should be performed in order to determine the definitive role of this new plate in treating the intra-articular olecranon fractures.

## Conclusions

The central tension plate with sharp hook contours to the anatomic morphology of the proximal ulna well. Treating intra-articular olecranon fracture with this plate can achieve good or excellent functional recovery with a high union rate and a low incidence of hardware related complications.

## Abbreviations

TBW: Tension band wiring; MEP: Mayo elbow performance; DASH: Disability of the arm, shoulder and hand questionnaire.

## Competing interests

The authors declare that they have no competing interests.

## Authors’ contributions

YZ and WC designed the study and wrote the manuscript. QZ, ZH and WC conducted the study, performed follow up, and assessed the functional outcomes of the affected limbs, under YZ’s supervision. QZ and ZH both helped to analyze data and revised the manuscript. All the authors agreed on the final content of the manuscript.

## Pre-publication history

The pre-publication history for this paper can be accessed here:

http://www.biomedcentral.com/1471-2474/14/308/prepub
